# Lower respiratory tract microbiome dysbiosis impairs clinical responses to immune checkpoint blockade in advanced non‐small‐cell lung cancer

**DOI:** 10.1002/ctm2.70170

**Published:** 2025-01-10

**Authors:** Yong Zhang, Xiang‐Xiang Chen, Ruo Chen, Ling Li, Qing Ju, Dan Qiu, Yuan Wang, Peng‐Yu Jing, Ning Chang, Min Wang, Jian Zhang, Zhi‐Nan Chen, Ke Wang

**Affiliations:** ^1^ Department of Cell Biology National Translational Science Center for Molecular Medicine Fourth Military Medical University Xi'an China; ^2^ State Key Laboratory of New Targets Discovery and Drug Development for Major Diseases Xi'an China; ^3^ Department of Pulmonary and Critical Care of Medicine The First Affiliated Hospital of Fourth Military Medical University Xi'an China; ^4^ Department of Microbiology School of Basic Medicine Fourth Military Medical University Xi'an China; ^5^ Department of Thoracic Surgery The Second Affiliated Hospital of Fourth Military Medical University Xi'an China

**Keywords:** advanced non‐small‐cell lung cancer, clinical outcomes, immune checkpoint blockade therapy, lower respiratory tract microbiome, multi‐omics analysis

## Abstract

**Background:**

Gut microbiome on predicting clinical responses to immune checkpoint inhibitors (ICIs) has been discussed in detail for decades, while microecological features of the lower respiratory tract within advanced non‐small‐cell lung cancer (NSCLC) are still relatively vague.

**Methods:**

During this study, 26 bronchoalveolar lavage fluids (BALF) from advanced NSCLC participants who received immune checkpoint inhibitor monotherapy were performed 16S rRNA sequencing and untargeted metabolome sequencing to identify differentially abundant microbes and metabolic characteristics. Additionally, inflammatory cytokines and chemokines were also launched in paired BALF and serum samples by immunoassays to uncover their underlying correlations. The omics data were separately analyzed and integrated by using multiple correlation coefficients. Multiplex immunohistochemical staining was then used to assess the immune cell infiltration after immune checkpoint blockade therapy.

**Results:**

Lower respiratory tract microbiome diversity favoured preferred responses to ICIs. Microbial markers demonstrated microbial diversity overweight a single strain in favoured response to ICI therapy, where Bacillus matters. *Sphingomonas* and *Sediminibacterium* were liable to remodulate lipid and essential amino acid degradations to embrace progression after immunotherapies. Microbiome‐derived metabolites reshaped the immune microenvironment in the lower respiratory tract by releasing inflammatory cytokines and chemokines, which was partially achieved by metabolite‐mediated tumoral inflammatory products and reduction of CD8^+^ effective T cells and M1 phenotypes macrophages in malignant lesions.

**Conclusions:**

This study provided a microecological landscape of the lower respiratory tract with advanced NSCLC to ICI interventions and presented a multidimensional perspective with favoured outcomes that may improve the predictive capacity of the localized microbiome in clinical practices.

**Highlights:**

- Alterations of the lower respiratory tract microbiome indicate different clinical responses to ICB within advanced NSCLC.
- Reduced microbial diversity of lower respiratory tracts impairs anti‐tumoral performances.
- Microbe‐derived metabolites perform as a dominant regulator to remodify the microecological environment in lower respiratory tracts.
- Multi‐omics sequencings of the lower respiratory tract possess the potential to predict the long‐term clinical responses to ICB among advanced NSCLC.

## Graphical abstract

1. Alterations of the lower respiratory tract microbiome indicate different clinical responses to ICB within advanced NSCLC.
2. Reduced microbial diversity of lower respiratory tracts impairs anti‐tumoral performances.
3. Microbe‐derived metabolites perform as a dominant regulator to remodify the microecological environment in lower respiratory tracts.
4. Multi‐omics sequencings of the lower respiratory tract possess the potential to predict the long‐term clinical responses to ICB among advanced NSCLC.

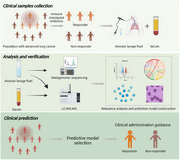

## 1 BACKGROUND

Immune checkpoint blockade (ICB), taking programmed death 1 (PD‐1) and its ligand as a typical example, has achieved impressive advancements on preferred therapeutic regimens of advanced NSCLC with promising clinical outcomes when compared with conventional strategies.^1^, ^2^ As the primary approved anti‐tumoral monoclonal antibodies towards PD‐1 by the American Food and Drug Administration (FDA) in 2015, Pembrolizumab (*Keytruda*) yields superior long‐term survival as a single‐agent intervention compared with platinum‐based chemotherapy arms in advanced NSCLC regardless of PD‐L1 proportion.^3^ However, restricted sufferers with advanced NSCLC may get enough therapeutic benefits from this scheme, while up to 30% experience drug‐ or dose‐related adverse events, where severe toxicities reach nearly 13.6% of patients receiving monotherapy.^4^ Restricted proportion of patients responding to ICB therapy aspires to an urgency, exploring precise biomarkers for screening out potential NSCLC sufferers with definite clinical efficacy, rather than mere reliance on PD‐L1 expression on tumour cells.

Recently, growing evidence have proved the favoured clinical outcomes mainly depend on cytokine‐ and chemokines‐driven CD8 positive T cell infiltration and the reduction of other suppressive immune cells in tumour microenvironment, which is accepted as an effective biomarker to predict the clinical efficacy of ICB interventions, except for those with high tumour mutation burden (TMB) and other neoantigens.^5^, ^6^, ^7^ Unfortunately, it seems impractical to launch dynamic monitoring based on tissue‐dependent invasive examination under the condition of the sustaining alterations of targeted effective T cell responses to neoantigens achieved from ICB.^8^ In contrast, electronic bronchoscopy examination accounts for series of advantages to indicate the immune characteristics of local tumour microenvironment by detecting inflammatory factors, lower respiratory tract microbiome, and corresponding metabolomic profiles.^9^ To date, despite restricted validations of the oncogenic capacity within biological components from the local microenvironment, the distinct correlation between local respiratory tract microecological environments and ICB response phenotypes still deserves additional attention.

Of note, biological significance of primary bacterial constituents in microbiome has been highlighted in modulating efficacy of ICIs in cancer. The distant microbiome in the gut fully discussed previously mediates diverse responses to immunotherapy due to its diversity and compositional differences, serving as potential predictive biomarkers and ongoing interventional strategies.^10^, ^11^, ^12^ Additionally, a series of studies further discovered the presence of intra‐tumoral and tumour‐resident microbiota mattered in cellular heterogeneity,^13^, ^14^ biological processes,^15^, ^16^, ^17^ and even immune microenvironment remodulation,^18^, ^19^ which has partially been materialized by engineered bacteria in immunotherapy.^20^ Mechanically, these microbiota mainly reshape the systemic immune responses by producing a set of dominant metabolites including bile acid, short chain fatty acids, and other microbiome‐associated mediators to promote effective T cell infiltration and recovery the tumoral immune balance.^21^, ^22^ However, microbiome in lower respiratory tract, which was found to be nonsterile in healthy conditions with boost development of metagenomic sequencing,^23^ fails to be elucidated in mediating response differences to ICB in NSCLC. Given this, it is still of significance to reveal the prospectives of lower respiratory tract microbiomes in prediction for the responses to ICB treatment and their underlying mechanisms of microbial metabolite‐mediated immune infiltration among patients with advanced NSCLC.

In this study, to identify lower respiratory tract microbiome and relevant metabolites when predicting responses to ICBs, we collected bronchoalveolar lavage fluids and paired serum samples from 26 patients with advanced NSCLC after receiving normative anti‐PD‐1 blockade monotherapy, a cohort of which was divided into three subgroups, including progressive disease, stable disease, and partial response groups according to indicated imaging features after standardized interventions. Then, 16S rRNA gene sequencing, and untargeted metabolome, together with integrated analysis, were performed to investigate the dominant genus that distinguishes between both groups. Besides, immunotherapy‐associated cytokines and chemokines detection in fluid samples and multi‐colour immunohistochemistry of primary immune cells in biopsy tissues were also conducted to evaluate the potential functions of microbiota‐mediated immune infiltrations in advanced NSCLC. Collectively, our study revealed comprehensive microecological profiles in different clinical responses to ICB monotherapy, further contributing to response prediction models using multi‐omics combined biomarkers as a relatively non‐invasive approach in the clinic.

## 2 METHODS

### 2.1 Cancer cohort enrollment and therapeutic outcomes evaluation

Patients with advanced NSCLC were recruited from our own single centre at the Department of Pulmonary and Critical Care Medicine, Xi‐Jing Hospital, First Affiliated Hospital of Fourth Military Medical University. Participants enrolled were at stage IIIB/IV according to TNM staging (Version 8th) and received immune checkpoint blockade monotherapy or combined regimes according to Guidelines for the Diagnosis and Treatment of Non‐Small‐Cell Lung Cancer (CSCO, 2022) and Clinical Practice Guidelines in Oncology Non‐Small‐Cell Lung Cancer (NCCN, 2022). After at least four cycles of the standardized treatment of indicated immune checkpoint inhibitors until regime alteration according to imaging features, all enrolled patients who refused to combine or failed to undergo chemotherapy were classified into partial response (PR, *n* = 4), stable disease (SD, *n* = 13), and progressive disease (PD, *n* = 11; 2 of them were deleted by failure during PCR amplification) mainly by their responses to anti‐tumour schemes as defined by response evaluation criteria in solid tumours (RECIST; version1.1), excluding those on pseudo‐progression and delayed immune treatment efficacy. Subjects were excluded if they had a history of antibiotics and glucocorticoid drug utilization in the past 3 months, combined respiratory acute or chronic infectious diseases, significant alterations in dietary habits within the previous 3 months, and other conditions in which they failed to perform immunotherapy normatively. All clinical information was collected according to standard procedures. All subjects provide written, informed consent for participation, and consented enrollment. This study was approved by the Ethics Committee of the First Affiliated Hospital of the Fourth Military Medical University (#XJYY‐LL‐FJ‐002). The Academic Integrity Supervision Committee of Fourth Military Medical University supervised entire procedures during conduction. All the procedures were adhered to the Declaration of Helsinki.

### 2.2 Bronchial‐alveolar lavage fluid and serum sample collection

The recruited population were performed bronchial‐alveolar lavage by electronic bronchoscopy examinations by the same professional physician in our department after efficacy assessments. Lavage fluids underwent preheated sterile physiological saline for enough volume, reaching no less than 60% as a stable recovery rate, and were collected into sterile containers, which were centrifugated at 4°C 12 000 rpm for 40 min. Centrifugal sedimentation and supernatant were segregated and restored at –80°C. Peripheral venous blood samples were sampled from the same patients into blood collection tubes without ethylenediaminetetraacetic acid on the morning before BALF collection. After adequate precipitation for at least 1 h at room temperature, samples would be centrifuged and separated at 4°C 3000 rpm for 10 min, then 200 µL per case of the supernatant was drawn and placed in numbered EP tubes and frozen at −80°C. All samples were applied for further analysis concurrently until processing as mentioned previously.

### 2.3 Gene sequencings and bioinformatics analysis

This section was achieved on Gene Denovo Co., Ltd. Briefly, bacterial DNAs were isolated from bronchial and alveolar lavage fluids (BALFs) samples using HiPure Stool DNA kits (D3141, Magentec). PCR amplification was sequentially launched on the 16S rRNA gene V3 and V4 hypervariable region using the primers 341F: CCTACGGGNGGCWGCAG, and 806R: GGACTACHVGGGTATCTAAT, respectively. QIAquick gel extraction kit (QIAGEN) was taken to purify PCR products, which were further quantified by Qubit 2.0 Fluorometric High Sensitivity dsDNA assay (Life Technologies), and then pooled due to their relative band intensity. Agencourt AMPure XP bead‐based reagent (Beckman Coulter Inc.) was also taken into consideration to combine purified PCR products following the manufacturer's protocol at steps. Then, high‐throughput amplicon sequencing was a necessity to conduct on the HiSeq platform (Illumina Inc) by using 2 × 150 paired‐end fragments.

16S rRNA gene sequencing was identified according to Software Quantitative Insights into Microbial Ecology. Operational taxonomic unit (OTU) counts per sample were generated and subsequently divided into taxonomies. Compositional and indicator species analyses were performed based on sequencing data. The biological diversities in the lower respiratory tract community were then achieved by alpha and beta diversity according to indicated compositional differences at corresponding levels, respectively.

### 2.4 Untargeted metabolomics sequencing and data processing

This section was achieved by Gene Denovo Co., Ltd. In brief, 100 mL BALF samples were resuspended with 400 µL prechilled 80% methanol. After incubation for 5 min, 0°C and contribution at 15 000 g, 4°C for 20 min, the supernatant was diluted to a final concentration containing 53% methanol by LC‐MS grade water. The samples were subsequently centrifuged at 15 000 g, 4°C for 20 min in fresh EP tubes, the supernatant of which was further injected on LC‐MS/MS system analysis. Details were shown as described in the previous study.^24^ The raw data files from the UHPLC‐MS/MS platform were identified on the Compound Discoverer 3.1 (CD3.1, ThermoFisher), to clarify peak alignment, peak picking, and quantitation for each metabolite. Primary settings were applied to the following: retention time tolerance to 20 s; actual mass tolerance for 5 ppm; signal intensity tolerance up to 30%; signal/noise ratio to 3; and minimum intensity for 100 000, aiming to normalize peak intensities into total spectral intensity. Additive ions, molecular ion peaks, and fragment ions were taken into account to predict the molecular formula according to normalized intensity as mentioned above. And then peaks were matched with the mzCloud (https://www.mzcloud.org/), mzVaultand Mass List database to obtain the accurate qualitative and relative quantitative results. Additionally, normal transformations were taken into account to optimize abnormally distributed data in light of the area normalization method.

### 2.5 Cytokines and chemokines detection

Cytokines and chemokines in lavage fluid and serum samples were detected by LEGENDplex bead‐based immunoassays (BioLegend) according to the manufacturer's instructions. The Human Inflammation Panel 1 (NO.740808, Biolegend) was used to analyze 13 human serum inflammatory cytokines/chemokines as described. Data acquisition was achieved on a BD FACSCalibur flow cytometer (BD Biosciences) and then analyzed with the LEGENDplex Data Analysis Software (BioLegend).

### 2.6 Multiplex immunohistochemical staining

Multi‐colour immunohistochemistry was performed to examine the candidate immune cell types in tissues. These tumour tissues were collected among the enrolled population by the time of diagnostic electronic bronchoscopy examinations following BALF sample collections. Those three paired tissues after ICI interventions were obtained from repeated electronic bronchoscopy examinations after standardized at least four‐cycle immune checkpoint blockade regimes according to the cohort enrollment scheme. The samples collected from the same patients undergoing electronic bronchoscope examinations in this cohort were fixed in time into 10% formalin for 48 h once upon clinical biopsy, which were further dehydrated and embedded in paraffin. In this practice, 4 mm slices were favoured to be sectioned into those paraffin‐blocked specimens, which were further affixed to glass slides heated at 70°C for 1 h. After those, slides were then deparaffinized in xylene and rehydrated in time within different alcohol concentrations, following 70%, 95%, and 100% in sequence. We further employed a multiplex immunofluorescence staining method to detect indicated cells on these slides with the help of the Opal 6‐Plex manual detection kit (NEL811001KT, AKOYA Biosciences). Primary antibodies were utilized as indicated concentrations (CD4, 1:50; CD8, 1:100; CD68, 1:1000; CD206, 1:400).

### 2.7 Quantification and statistical analysis

Bioinformatic analysis was further conducted by means of Omicsmart online platform (see also Additional file 1). Correlations between clinical responses with clinical information were analyzed by Fisher's test as mentioned in Supporting Information Files, several of which were also presented as means ± SEM or SD. Nonparametric Mann–Whitney was performed to identify the statistical significance of the relative abundance of OTU counts, diversities, and immune parameters within indicated subgroups. Univariate survival analysis based on progression‐free survival was performed by log‐rank test according to indicated data types. The Cox proportional hazards model using forward stepwise selection was conducted to further identify independent prognostic factors associated with PFS. Pearson linear and Spearman rank‐sum correlations were also achieved to explicit the correlations between cytokines and chemokines in BALFs and serums. Statistical analyses were performed using Microsoft Excel (Microsoft Inc.), GraphPad Prism 9.0 (GraphPad Software Inc.), and Origin (Origin Software Inc.). Results were analyzed with two‐tail, reaching statistical significance if the *p*‐value was less than .05. Scientific figures were drafted by BioRender software online as mentioned in Additional file 1.

## 3 RESULTS

### 3.1 Cohort characteristics and clinical responses to ICB

**FIGURE 1:**
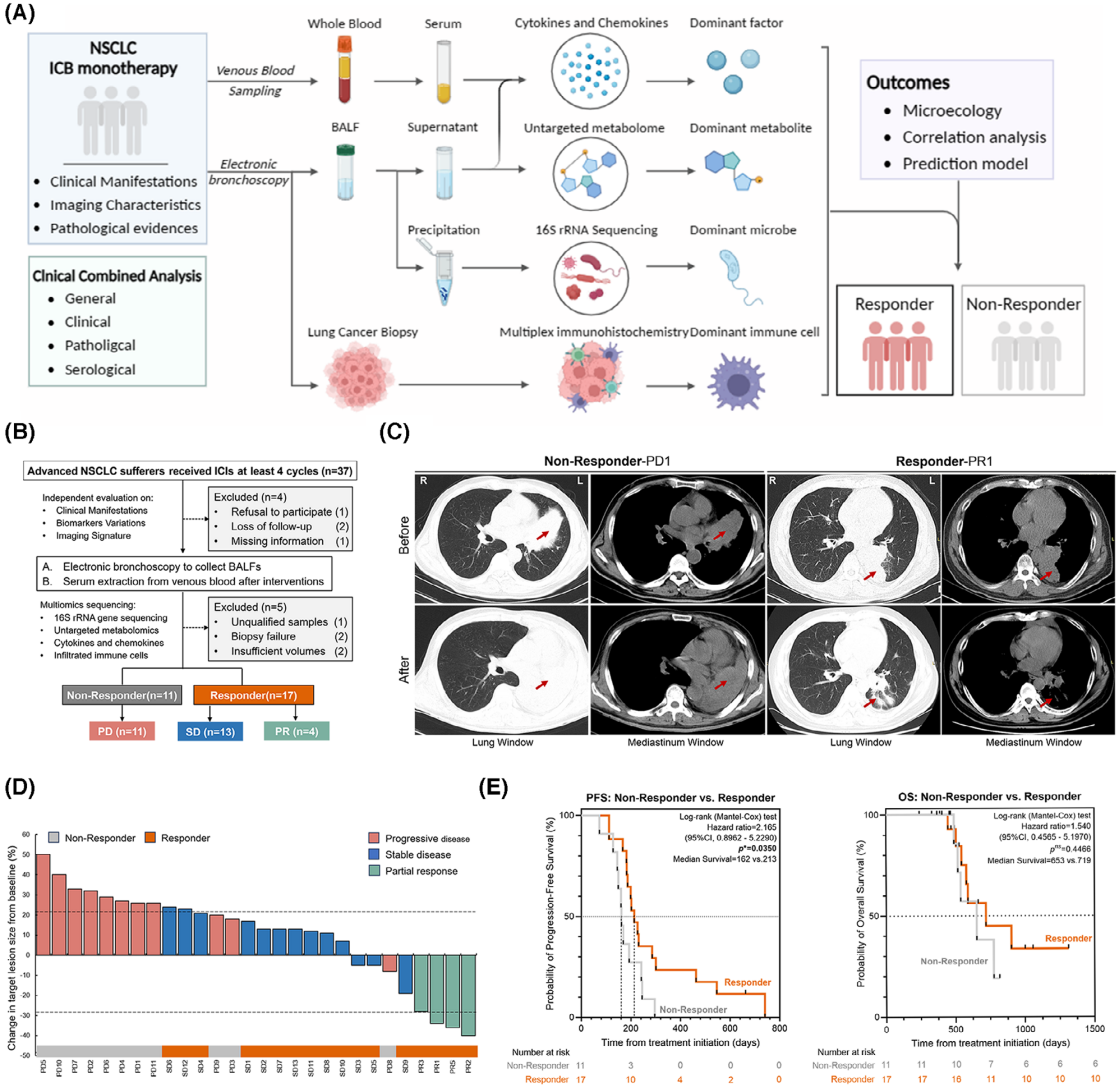
General design of this study and clinical characteristics of enrolled cohorts. (A) Flow chart of this study. (B) Workflow demonstrating the cohort enrollment and exclusion criterion. Subsets are divided into non‐responder (NR) and responder (R) groups, or progression disease (PD), stable disease (SD) and partial response (PR) according to RECICST 1.1. (C) Representative Computed Tomography (CT) scanning images of indicated participants before and after immune checkpoint blockade interventions at the identical layers among the same patients, respectively. Lung and mediastinum windows are shown as marked. Red arrow, suspected lesion sites. (D) Best changes in the aggregate diameter of target lesions by mRECIST. Dashed lines represent a 20% increase in disease progression (up) and a 30% decrease due to partial response (down). (E) Kaplan–Meier plot comparing the progression‐free survival (left) and overall survival (right) of responders and non‐responders recruited in this study. Dashed lines represent indicated survival reached 50%, respectively. Detailed information is listed on the upper right correspondingly. See also Figure S2.

Accordingly, microecological environment characteristics in the lower respiratory tract were collected from patients suffering from advanced NSCLC receiving immune checkpoint inhibitors with available information on response to ICIs in a retrospective single‐centre cohort (Figure 1A). In detail, samples collected after the standard single‐agent therapeutic ICI regimes without antibiotics and other interventions were involved to focus on the potential microbial and metabolic elements for ICI response prediction, mainly from 28 patients in this study divided into non‐responder (NR, *n* = 11) and responder (R, *n* = 17) or progressed disease (PD, *n* = 11), stable disease (SD, *n* = 13), and partial release (PR, *n* = 4) according to Response Evaluation Criteria in Solid Tumors 1.1 evaluations, baseline manifestations of which were nearly consistent to compare (Figure 1B; Table S1). Abiding by clinical performances, these categorizations were predominantly achieved in accordance with the imaging characteristics compared with naïve malignant sites, as representative CT scanning was shown to evaluate responses to ICIs after regular and adequate interventions, respectively (Figure 1C). The primary malignant lesion sizes varied from each baseline after receiving ICI regimes, in line with the imaging characteristic‐based response evaluation, further supporting the accuracy of CT scanning‐based classifications in this study (Figure 1D). Another, progression‐free survival within each subgroup also indicated the positive outcomes in the Response group, which failed to achieve in non‐response instead (^*^ *p *< .05), while the overall survival was far to reach, partially due to a shortage of extended follow‐ups and restricted cohort populations (Figure 1E; Table S2). These traits illustrated the effective and accurate categorizations of the enrolled cohort population in this study, facilitating the coming sequencings and analysis in depth.

### 3.2 Differentiated abundance of *Bacillus* associated with response to ICI therapy

**FIGURE 2:**
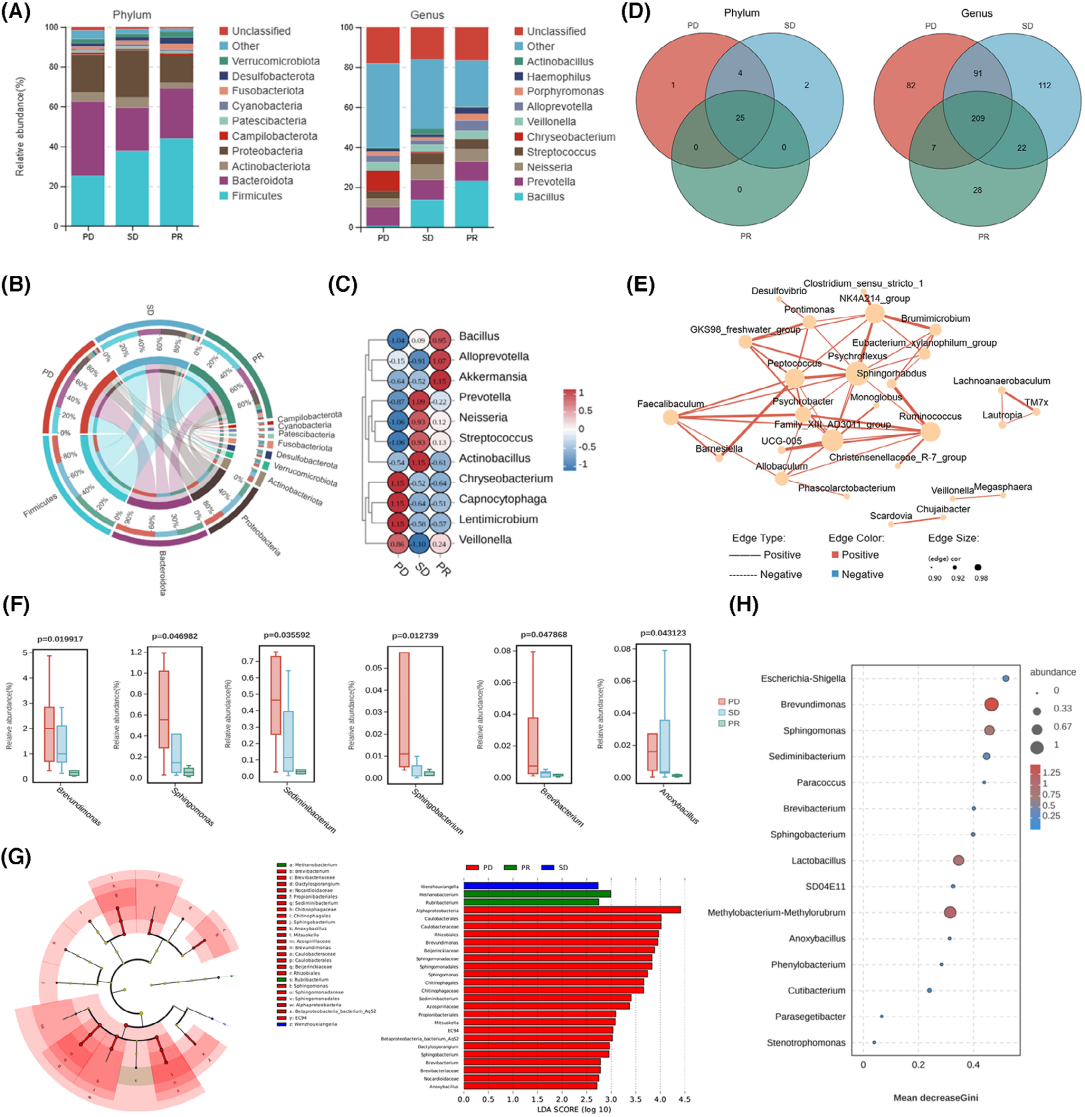
Microbial profile alterations among lower respiratory tracts to ICB response in advanced NSCLC. (A) Microbial taxonomy at phylum (left) and genus (right) level with indicated subgroups. (B) Circos plot displaying the relative abundance of microbial phylum compositions over .1%. (C) Heatmap of top dominant genera based on relative abundances at different subgroups. *p*‐values of genera are listed at corresponding sites. Colour gradients represent the value of degree of deviation. (D) Venn diagram of the observed phylum (left) and genus (right) in indicated subgroups. (E) Co‐occurrence network of microbial markers in ICB responders and non‐responders. Size of each node is proportional to the mean relative abundance. The 95% credible interval criteria were used to assess significance, and estimated correlations were then filtered with the correlation coefficient≥.4 by Spearman correlation analysis at genus level. Positive correlations were coloured in red with a solid line and negative in blue with a dashed line. (F) Bot plots display the prevalence of the relative abundance for the most prevalent genera (present at≥3% in any one of the samples), illustrating the dissimilarity in the microbial composition of each group. Significance was determined using the Kruskal–Wallis rank‐sum non‐parametric test on each plot (*p *> .05 not shown). (G) Cladograms showing microbial compositional differences at indicated subgroups analyzed by linear discriminant analysis effect size (LEfSe) method. From domain to species with linear discriminant analysis score (LDA) > 2. (H) Random forest showing the indicating genera among PD vs. PR based on mean decrease Gini. The size and colour of bubbles represent the abundance of corresponding genera, while the position of bubbles represents the size of the index. See also Figure S3.

In order to reveal microbial components of lower respiratory tract microbiome on positive response to ICI agents, aseptic processed bronchoalveolar lavage fluids from enrolled participants by response phenotypes were put into 16S rRNA sequencing, aiming to screen out the dominant microbes and quantify the effects of potential confounders. After striking out two undetectable ones during PCR amplification, microbial compositional analysis from 26 samples showed that the dominant microbes altered in response to ICIs with the increasing malignant controls at phylum and genus levels, respectively, mainly which featured the *Bacillus* overweighting and *Chryseobacterium* shrinking (Figure 2A) in accordance with that in R and NR groups (Figure S1A,B). It is still worth noting that *Firmicutes* seemed associated with positive responses to ICI interventions, while *Bacteroidota* accounted for a relatively large proportion at the phylum level among unfavourable outcomes (Figure 2B,C; Figure S1B). Although the presence of overlapped candidates, distinctive microbes within indicated subgroups might also contribute to selective potencies to predict clinical outcomes of ICIs monotherapy at different levels (Figure 2D). As integrated occurrences in a sophisticated microecological environment, the interactions of these microbes reshaped quite unidentical microenvironments in lower respiratory tracts among different responses to ICIs within advanced NSCLC. SparCC was performed to calculate the correlations between indicated microbial markers, further identifying their underlying interactions, finding that *Sphingorhabdus* and other genera performed dominant interactions in lower respiratory tract microbiome no matter which responses were attributed to (Figure 2E; Figure S1F). To sift the positive response‐preferred microbes in local microecology, we attempted to launch the Kruskal–Wallis rank‐sum nonparametric test among these three groups, further identifying six lower respiratory tract bacteria with differential abundance between unidentical responses to ICIs at baseline (*p *< .05 at genera level) (Figure 2F), the majority of which were enriched in PD groups and partially consistent with the identified candidates by LEfSe (Figure 2G; Table S3). Indeed, the relative enrichment of differentiated candidates, including *Brevundimonas*, *Sphingobacterium*, and other bacteria in PD, has been proven to correlate with oncogenesis and unexpected outcomes in several cancer types,^25^, ^26^, ^27^ partially supporting our findings theoretically, which was well displayed by random forest analysis in detail (Figure 2H; Table S4). These results pointed to a unique value that the distinguishable performances of microbes in localized environments benefit the responsive prediction before the ICI initiation in advanced NSCLC.

### 3.3 Lower respiratory tract microbiome diversity favoured preferred responses to ICIs

**FIGURE 3:**
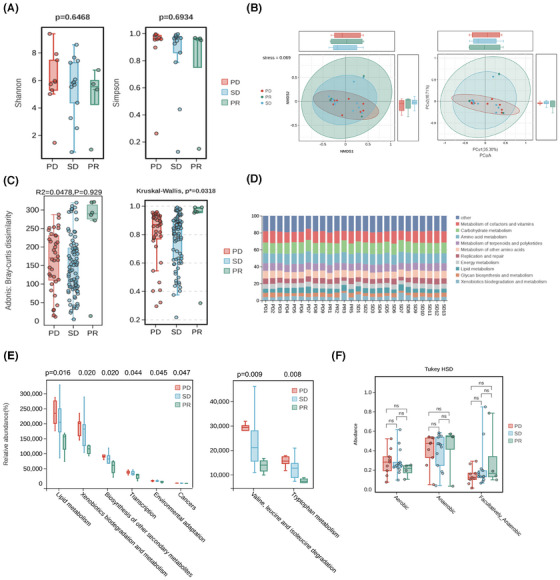
Species diversity analysis and microbial functional discrepancies of gene expressing in responses to ICB monotherapy. (A) Alpha diversity measured by inverse Shannon (left) and Simpson (right) index using the Kruskal–Wallis rank‐sum non‐parametric test. *p*‐values are shown on the top. (B) Non‐metric multidimensional scaling (NMDS, left) and principal Coordinate analysis (PCoA, right) of beta diversity measurements based on Bray‐Curtis distances for PD, SD, and PR. Each symbol represented one individual patient. (C) Adonis (permutational MANOVA, Permanova, left) and indicated statistical analysis (right) showing the outcomes of beta diversity measurements based on Bray‐Curtis distances for PD, SD, and PR via the Kruskal–Wallis rank‐sum non‐parametric test. *p*‐values are shown on the top. (D) Stacking diagram showing the top 10 function alterations of each patient at level 2 in PICRUSt2 database. (E) Box plots showing the differential pathways at level 2 (left) and level 3 (right) within indicated subgroups based on relative abundances using the Kruskal–Wallis rank‐sum non‐parametric test. *p*‐values are shown on the top. The plot depicts the median value, interquartile range, and 95% confidence intervals. (F) Comparison of oxygen preferences of microbial compositions within indicated subgroups based on BugBase database using Tukey's honest significant difference (HSD). Each symbol represented one individual patient. Ns, no significance. See also Figure S4.

Regarding the landscape of the lower respiratory tract microbiome, the diversity seemingly matters most in explaining the microbial compositional prediction values rather than a single microbe instead. Notably, alpha diversity of the lower respiratory tract microbiome failed to reach significance by Shannon and Simpson index regardless of different clinical outcome‐oriented classifications (Figure 3A; Figure S1C), mainly contributing to restricted cohorts enrolled in this study, while the tendencies did remain. Another, Bray‐Curtis distances dependent beta diversity analysis showed that the presence of significances between the indicated groups featured relatively unique compositions among different responses to ICIs according to NMDS (stress = .069) and PCoA, respectively (Figure 3B; Figure S1D,E). It was also found in Adonis analysis that an optimized testing performance adhered to different ICI responses among the indicated groups (Figure 3C), further indicating a distinguishable performance of lower respiratory tract microbiome in predicting ICI responses within advanced NSCLC participants.

Of note, microbial discrepancy also contributed to alienated metabolic pathways in hosts as resulting or reasoning reactions to microbial alterations. Generally speaking, subjects enrolled in different groups shared similar metabolic pathways (Figure 3D; Figure S2A). Liquid metabolism pathway (^*^ *p *= .016), xenobiotics biodegradation and metabolism pathway (^*^ *p *= .020), and biosynthesis of other secondary metabolites (^*^ *p *= .020) at Level 2, and valine, leucine, and isoleucine degradation (^**^ *p *= .009) and tryptophan metabolism (^**^ *p *= .008) at Level 3 (Figure 3E) characterized significances among these indicated groups, which were reported to be associated closely with candidate microbes mentioned above in our study and functioned in modulating the immune microenvironment to ICI responses (Figure S1G,H and S2C). These metabolic alterations might be achieved by dominant microbe‐mediated bacteria–host interactions and metabolic remodulation to some extent. Additionally, there seemed no significance in oxygen preference among different ICI responses, while the tendencies that the proportion of facultatively anaerobic microbes escalated and that of aerobic ones descended were also noticed (Figure 3F), partially pointing that oxygen intake may reshape the microbial composition and benefit the microbe‐dependent immunoregulation to key checkpoint blockade as former reported.28

### 3.4 Metabolic signatures and integrated analysis with microbiome reveal microecological landscape in lower respiratory tract

**FIGURE 4:**
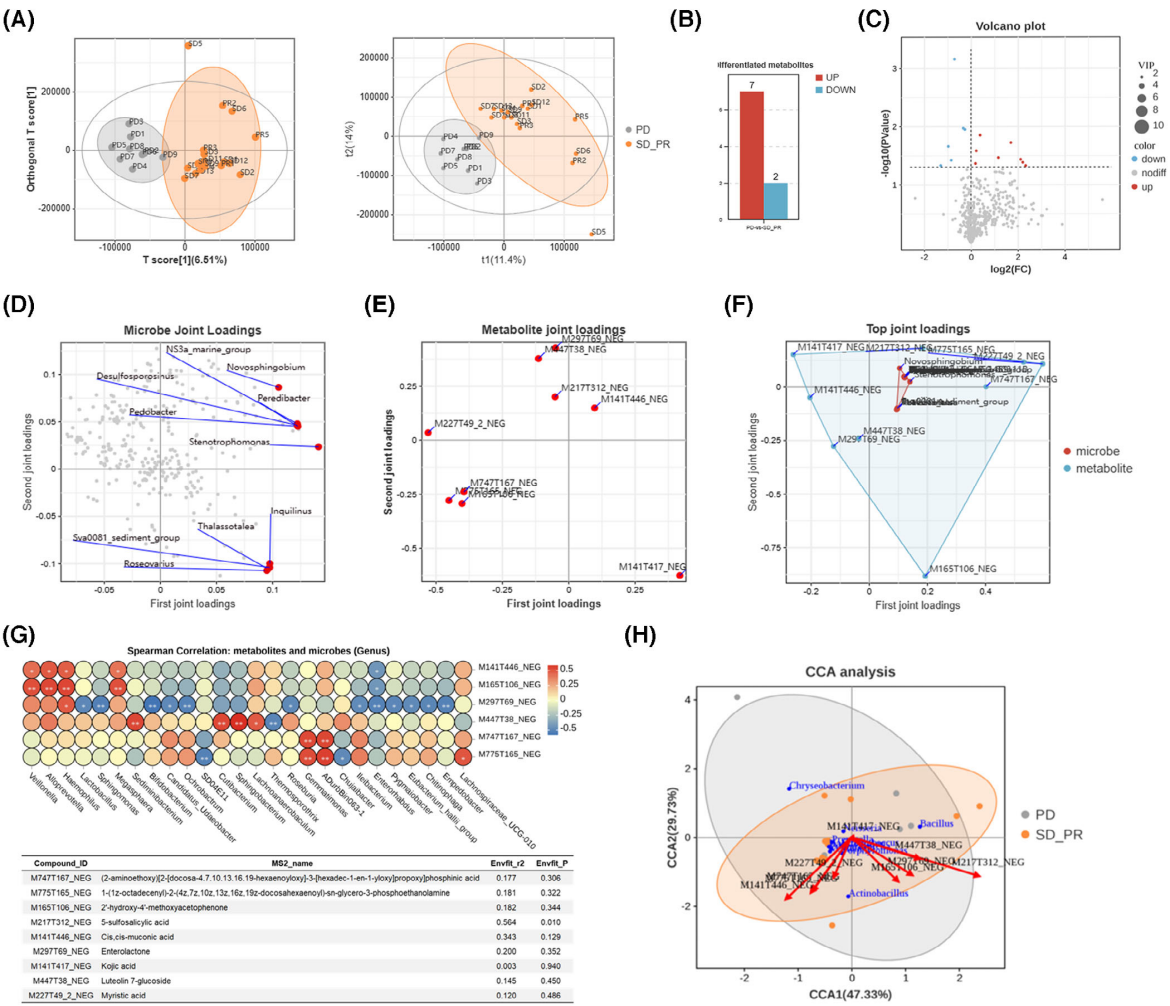
Untargeted metabolite profiles and potential associations with differentially enriched microbes among indicated subgroups. (A) Orthogonal partial least‐squares discriminant analysis (PLS‐DA, left) and partial least‐squares discriminant analysis (PLS‐DA, right) score plots of metabolomics data. Each symbol represented one individual patient. (B) Histogram of differentiated untargeted metabolites among NR and R (VIP≥1, ^*^ *p *< .05). (C) Volcano plot showing the differentially enriched metabolites. The horizontal axis represents the difference in multiple metabolite abundance in each comparison group, taken as log_2_. The vertical axis represents the *p*‐value after the *t*‐test, taken as −log_10_. The dashed line perpendicular to the *y*‐axis represents the *p*‐value threshold for screening differential metabolites (VIP≥1, ^*^ *p *< .05). Each symbol represents one individual patient. Red, upregulated metabolites with fold change (FC) > 1. Blue, downregulated metabolites with FC ← 1. Grey, not meeting the threshold screening. (D) Microbial load diagram showing top 10 genera with high contribution to microbial communities. The red dot represents the dominant genus and the grey dot indicates no significant microbes. (E) Metabolic load diagram showing the top 10 metabolites with a high contribution. The red dot represents the dominant metabolites with Compound IDs and the grey dot indicates no significant ones. (F) Microbial‐metabolite load diagram showing the distributed relations of dominant microbes and metabolites with high contribution at two joint loadings based on bidirectional orthogonal projections to latent structures (O2PLS) model. Red, microbe. Blue, metabolite. (G) Heatmap (top) showing the two‐tailed Spearman's rank correlation of candidate metabolites and microbes at genus level based on differential analysis. Significance on each point with an asterisk. ^*^ *p *< .05, ^**^ *p *< .01. Compound IDs equal to MS2 name are listed in an integral table (bottom). (H) Canonical correspondence analysis (CCA) biplot indicating the candidate microbial species and metabolites in NR and R. Each microbial sample is marked blue in the plot. Red arrows show the corresponding metabolites. See also Figure S5.

Except for biofilm‐directed contacts with host cells, exploiting nutrients or intermediate metabolites from the host to carry out biosynthesis and degradation seemed to be a priority to generate microbe–host interactions as a ubiquitous strategy. Unlike gut microbes mediated systemic influences by gut microbes, the lower respiratory tract microbiome inclined to highlight reshaping the localized microenvironment in a stable and lasting period of time despite slight microbial alterations within each dynamic gas exchange. However, microbiome‐associated metabolic characteristics were still retained no matter what the composition it possessed due to the relatively persistent presence of colonizing microbes. To this point, we identified the metabolic features of bronchoalveolar lavage fluid from enrolled cohort by untargeted metabolome, and further performed the integrated analysis, exploring the potent correlations between microbes and their metabolites in mediating ICI responses (Figure S3A). Surprisingly, it seemed quite different between R and NR as to the orthogonal partial least‐squares discriminant analysis scores and partial least‐squares discriminant analysis (PLS‐DA, right) scores, respectively (Figure 4A; Figure S3B,C), further indicating relative excellent predictive capacity to distinguish ICI responses validated by paired accuracy permutation tests. The variables with the dominant principal component contributions to the scores above were also verified according to the load diagrams. Additionally, differentiated metabolites were also indicated by variable importance for the projection (VIP) values (VIP > 1; ^*^ *p *< .05), identifying candidates with seven upregulated and two downregulated metabolites in bar and volcano plots (Figure 4B,C).

To further explore the potential orientations of key metabolites from the candidate microbes, we performed bidirectional orthogonal projections to the latent structures (O2PLS) model to identify the indicated correlations. Correspondingly, as the dominant correlated microbes and metabolites identified by calculating the sum of loading square values separately and integrated (Figure 4D–F; Figure S3D), lipid and amino acid‐related metabolites grasped close dependence to the differentiated genus microbes at the top 10 level due to relative extended distance to the original point. It should also be highlighted that these candidate metabolites correlated closely with variable microbes in the lower respiratory tract regardless of responses to ICIs to some point (Figure 4G; Figure S3E), which have been marked below and further confirmed by CCA analysis at the genus level as mentioned (Figure 4H). Interestingly, we found that five metabolites among integrated analysis overlapped with those in single‐omics metabolome, including luteolin 7‐glucoside, and enterolactone, most of which were consistent with results in Figure 3E. Collectively, these results showed correlations of candidate metabolites with microbes, the latter of which might be the microbial origins to produce these metabolites in reshaping the immune microenvironment in lower respiratory tracts, further remodifying the responses to ICIs to the end.

### 3.5 Microbiome‐derived metabolites reshaped immune microenvironment in lower respiratory tract by releasing inflammatory cytokines and chemokines

**FIGURE 5:**
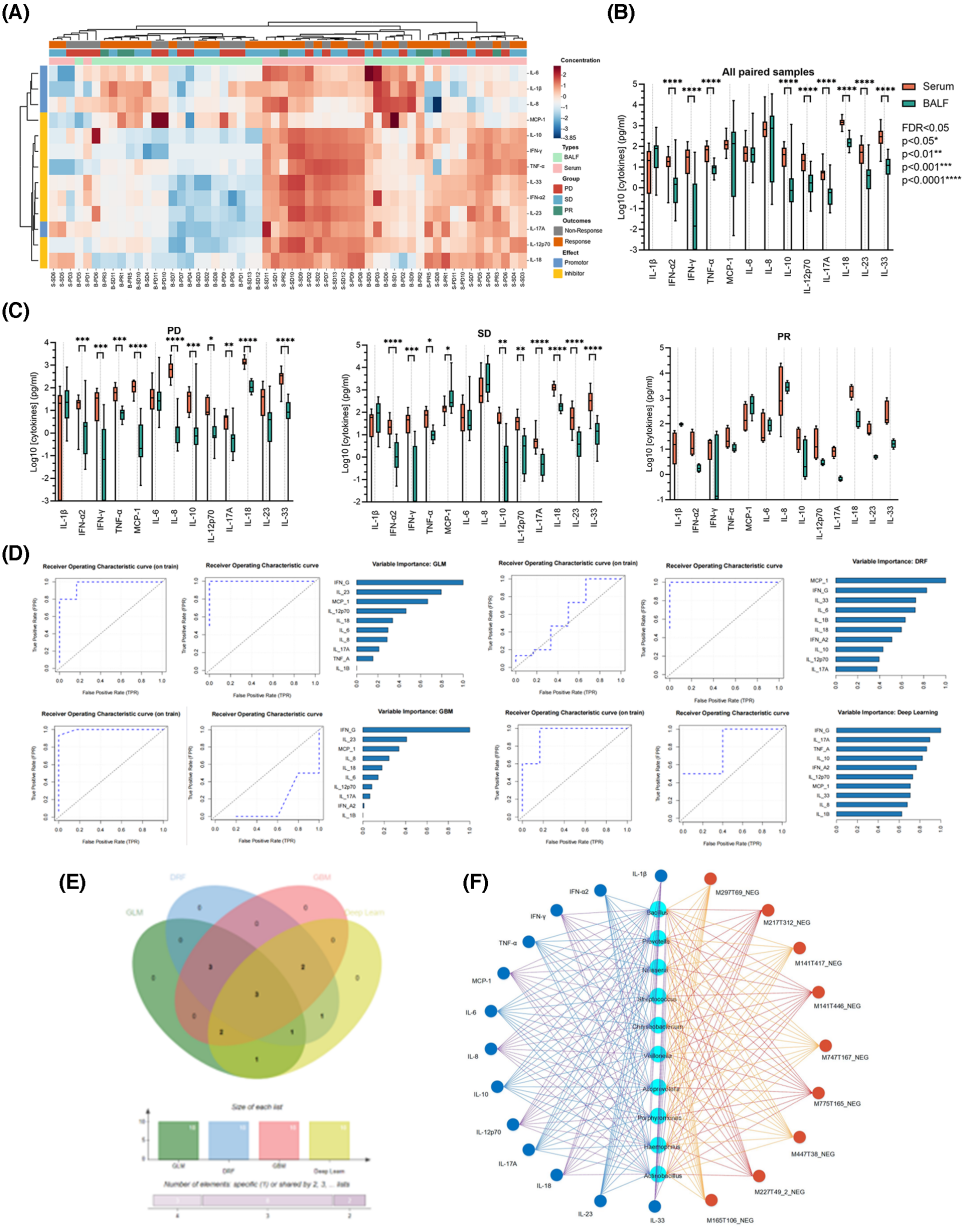
Compositional and correlational analysis of primary cytokines and chemokines in lower respiratory tract and serum. (A) Heatmap of detected concentration (log_10_FC, pg/mL) of tumoUr immunity‐related cytokines and chemokines via LEGENDplex bead‐based immunoassays. Details are shown in the Methods section. Cytokines and chemokines are divided into promoter and inhibitor subgroups by their corresponding correlation with tumour development as previously mentioned. (B) Box plot showing the diverse compositional differences of indicated cytokines and chemokines in all serum and BALF samples via LEGENDplex mentioned above. Relative *p*‐values are marked on the indicated paired groups with mean and standard deviation. FDR < .05, ^*^ *p *< .05, ^**^ *p *< .01, ^***^ *p *< .001, ^****^ *p *< .0001. No significance is shown. FDR, False discovery rate. (C) Box plots showing the diverse compositional differences of indicated cytokines and chemokines in serum and BALF samples among PD (left), SD (medium), and PR (right) subgroups via LEGENDplex mentioned above. Relative *p*‐values are marked on the indicated paired groups with mean and standard deviation. FDR < .05, ^*^ *p *< .05, ^**^ *p *< .01, ^***^ *p *< .001, ^****^ *p *< .0001. No significance is shown. (D) Receiver operating characteristic (ROC) curves (left) showing the AUCs of primary cytokines and chemokines in BALFs via 4 models with standard deviation of stratified 10‐fold cross‐validation, including generalized linear model (GLM), Gradient boosting machine (GBM), distributed random forest (DRF), and deep learning model, respectively. (E) Petal diagram showing the overlapped candidates among BALFs in NR and R subgroups with IFN‐α2, TNF‐α, and IL‐1β. (F) Network representing significance and stability selected correlations of differentiated microbes (bright blue), metabolites (red), and cytokines (blue) at FDR < .25 according to LASSO‐based differentiated analysis in the lower respiratory tract. See also Figure S6.

The specific microecological environment in the lower respiratory tract features distinctive microbial characteristics with different therapeutic outcomes to ICIs as discussed above. Mechanically, inflammatory factors, including cytokines and chemokines, are inclined to be generated with the presence of microbiome and its related metabolome collectively from tumours themselves, or more possibly, the immune cells. Thus, we launched a flow cytometry‐based multi‐analyte inflammation panel detection to identify the content expression of key cytokines and chemokines that correlated closely with anti‐tumour immunity in the lower respiratory tract and serum. Being consistent with previous reports, the expression of indicated inflammatory panels in BALF samples were at a relatively lower level than pared those in serum within both groups (Figure 5A; Figure S4), which could be mainly attributed to the restricted resident inflammatory cellular components. Although differences between both sampling sites did exist, it was of note that several inflammatory factors were enriched in BALF but lower than those in serum, such as IL‐1β, MCP‐1, and IL‐8, further indicating that immune cells in the lower respiratory tract or lung‐resident immune cells drove the specific inflammatory microenvironment formation, and correspondingly, reconstructed the diverse responses to ICI interventions, regardless of no significance in each sample dependent subgroups, either (Figure 5B; Figure S5A,B). Furthermore, we analyzed the relative expression of each candidate from both samples within different clinical response groups to explore the dominant roles of indicated inflammatory components in mediating ICI responses. Surprisingly, inflammatory candidates in PD were prone to overlap with those in the total, while the escalated cytokines, including IL‐1β, MCP‐1, and IL‐8, seemed to overweight those in serum among SD and PR subgroups of the Response cohort (Figure 5C), together with the Pearson linear regression (Figure S5C; Table S5) and spearman rank sum correlation (Table S6), which partially indicated a potential that lung‐resident immune cells might be the primary origins of these inflammatory factors, and in turn induce cytokines and chemokines mediated immune reconstruction as replies to ICI regimes, although some of them showed no significance to some extent (Figure S5D). The predictive values of these detected inflammatory candidates in BALF were also emphasized by receiver operating characteristics curves (ROC curves) generated by four types of machine‐learning approaches, including generalized linear model, gradient boosting machine, distributed random forest, and deep learn (Figure 5D). Although the diverse presence of area under the ROC curve, the overlapped Venn diagram pointed to a three‐components dependent predictive model with IL‐1β, TNF‐α, and IFN‐α2, which were also achieved in serum with five candidates instead (Figure 5E), indicating relative effective predictive values of ICI interventions by inflammatory examination from the lower respiratory tract. We also noticed the potent correlations between the sifted microbes, metabolites, and indicated inflammatory penal (Figure 5F), the whole of which depicted the extrapulmonary inflammatory environment and further presented a microbe‐dependent metabolite‐driven cytokines and chemokines releases in modulating ICI responses in advanced NSCLC.

### 3.6 Effective T cells and macrophage infiltration performed as cellular biomarkers to preferred anti‐tumour immune outcomes

**FIGURE 6:**
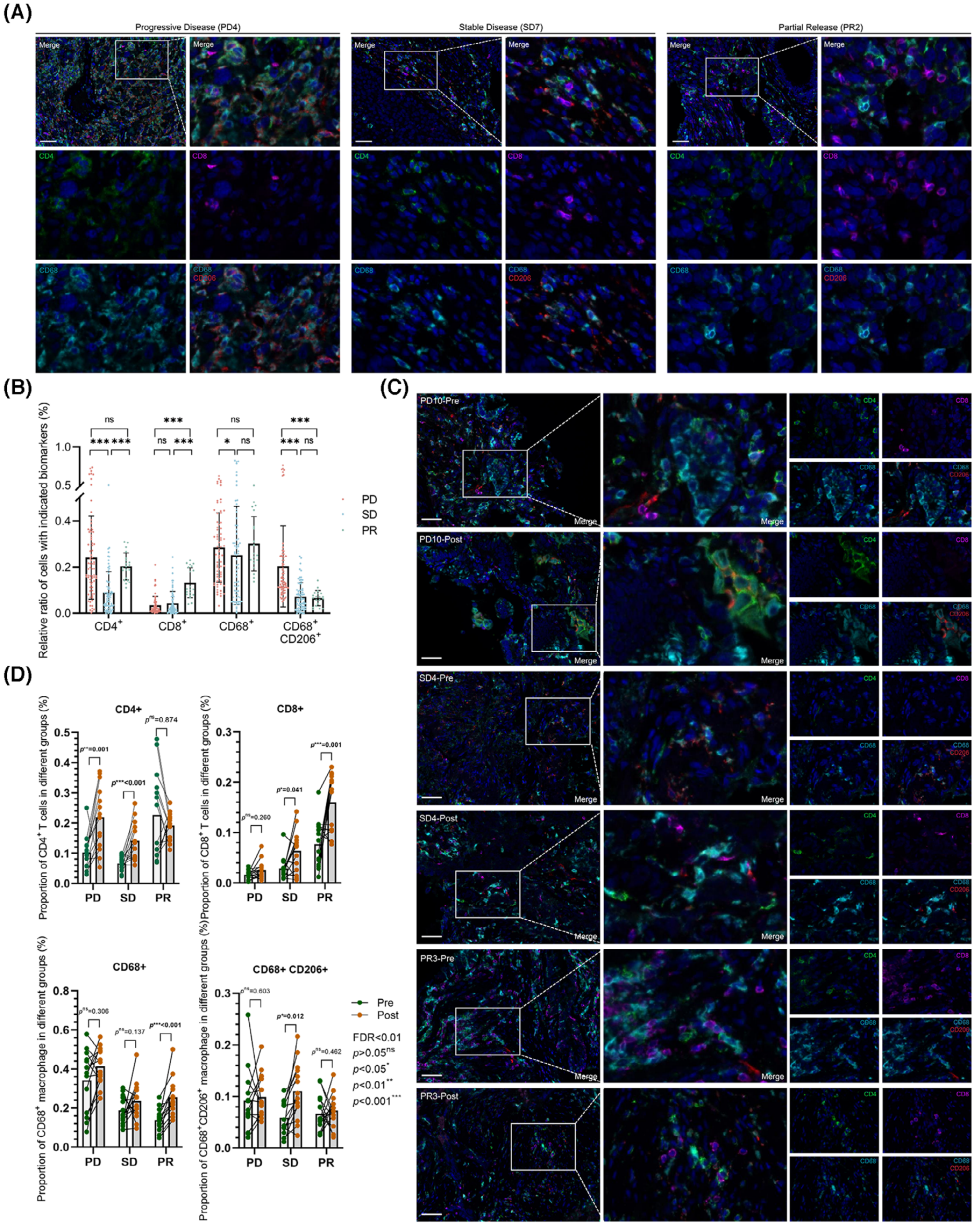
Anti‐tumour immune cell infiltration of different responses to ICB in advanced NSCLC. (A) Representative images of multi‐colour immunohistochemistry in diverse responses to ICB showing detection of DAPI (blue), CD4 (green), CD8 (purple), CD68 (cyan), and CD206 (red)‐positive cells. Scale bar, 50 µm. (B) Box plot showing the relative ratio of indicated biomarkers on immune cells within subgroups, including single CD4^+^, single CD8^+^, single CD68^+^, and CD68/CD206 dual positive immune cells, by Wilcoxon rank‐sum test (FDR < .01; ^*^ *p *< .05). Significance is shown as ^*^ *p *< .05, ^**^ *p *< .01, ^***^ *p *< .001, ns, no significance. (C) Representative images of multi‐colour immunohistochemistry in diverse responses to ICB within the same patient pre‐ and post‐ anti‐tumour immune interventions showing detection of DAPI (blue), CD4 (green), CD8 (purple), CD68 (cyan), and CD206 (red)‐positive cells. Scale bar, 50 µm. (D) Box plot showing the relative ratio and correlation of indicated biomarkers on immune cells between pre‐ and post‐receiving standard ICB monotherapy within indicated subgroups by Wilcoxon rank‐sum test (two‐stage step‐up, FDR < .01, ^*^ *p *< .05). Significance is shown on the corresponding sites as ^*^ *p *< .05, ^**^ *p *< .01, ^***^ *p *< .001, ns, no significance.

As the primary origins of anti‐tumorous immune mediators in the lung, besides the resident immune cells, adapted immune components were motivated by diverse stimuli with content alterations and functional dynamic regulations, from the bloodstream, colonized microecological environments, or both. In this circumstance, immune checkpoint blockade‐based therapeutic interventions tend to reshape the immune microenvironment or just perform as intermediators between localized microecological factors and clinical outcomes. Based on the results above, we next explored the immune constitution in enrolled tissues with advanced NSCLC after ICI interventions by multiplex immunohistochemical staining, which were illustrated as the dominant sources of inflammatory candidates as mentioned. In this section, primary biomarkers of effective T cells and tumour‐associated macrophages (TAMs) were stained in all patients enrolled in this cohort, exploring the constitutional differences between the diverse clinical outcomes with seven repeated samplings at random sites on the same slides (Figure 6A). No doubt was there that CD8‐positive effective T cells escalated and CD68‐CD206 double‐positive M2 macrophages descended along with improving ICI responses as previously reported, while the CD4 positive T cells and the total macrophages reached no significant differences between PD and PR (Figure 6B). Further, we performed identical approaches in retrospectively collecting two‐paired biopsy tissues before and after ICIs in each subgroup, aiming to detect these immune cell transformations after the utilization of ICIs (Figure 6C). Just as presumed, CD4‐positive T cells seemed less significantly variable in PR subgroups, which was upregulated in the other two groups. On the contrary, the proportion of anti‐tumorous CD8 positive T cells boosted as the therapeutic outcomes improved. Additionally, CD68 positive TAMs were set at a relatively stable platform in PD and SD, which slightly overweighted in PR as a response to ICIs, but transferred in M2 macrophages (Figure 6D), indicating that in patients with favoured clinical outcomes, M1 phenotypes accounted for a large majority of TAMs to exert anti‐tumour effects as a distinguishable biomarker in ICI response prediction. These results further illustrated that microbial components in the localized microecological environments from the lower respiratory tract may regulate anti‐tumorous immune effects by mediating cytokines and chemokines related immune cell infiltration or migration, which indicated an applicable predictive model to ICIs as well as a description of favourable outcomes in the lower respiratory tract.

## 4 DISCUSSION

Unsatisfactory outcomes of immune checkpoint blockade therapies restricted their clinical applications in advanced NSCLC. Although tumour or immune cells directed precise genomics diagnosis,^29^ including tumour mutation burden,^30^, ^31^ and PD‐1/PD‐L1 examinations,^32^, ^33^ have been extensively accepted as the preliminary explanations for responsive evaluations to ICIs, the presence of primary or acquired resistance to them still conflicts with the practical effectiveness of anti‐tumour immune therapies.^34^ Due to being embedded in specific microenvironments in the lower respiratory tract, NSCLC cells are liable to be influenced by localized various biological components, including colonized or translocated immune factors, normal and malignant host cells, and, of course, differentiated microbiomes.^35^ Recent research has highlighted the significance of gut microbiome in evaluating ICI responses,^36^, ^37^, ^38^ but these microbial biomarkers were quite unidentical upon geographic distributions, routine diets intake, human genetic features, and other petty elements by latent means.^12^ Even though the integrated analysis of bioinformatics narrows the gap between the sequencing data and clinical practices during ICI response predictions to some extent, the performance of indicated microbial biomarkers remains vulnerable to the genuine states. Most recent studies have discussed the dominant role of intra‐tumour bacteria in modulating biological progressions in lung cancer, especially the predictive performance to immunotherapies,^39^ but no similar attempts have been made in their dominant fountainhead, the microbiome from lower respiratory tracts.

In this study, we launched a retrospective single‐centre sampling approach to uncover the localized microecological characteristics in responders (R, equal to SD plus PR) and non‐responders (NR, equal to PD) to ICIs on advanced NSCLC, and subsequently explored underlying microbiome–metabolome–inflammatory panel–immune cell correlations from the four perspectives, aiming to uncover its predictive performance in ICI responses among advanced NSCLC. Correspondingly, we employed multi‐omics analysis approaches to illustrate the protective role of *Bacillus* and its relative lipid and amino acids metabolites, inducing MCP‐1 and other beneficial cytokines and chemokines release to recruit effective T cells and macrophages, mainly M1 phenotype, to further improve ICI responses, which pointed to a potential biomarker to evaluate ICI performance before its initiation. This highlights the pivotal role of the lower respiratory tract microbiome and localized microecological features in the context of ICI responses in the clinic.

Outbursts of deep sequencing and microorganism identification technologies give a preference to exploring microbe‐mediated dysfunctions from specific sites with low biomass used to be taken as aseptic for years, including lower respiratory tract microbiome.^40^ Lower respiratory tract used to be taken as sterile in normal conditions, which has been overthrown gradually, revising the opinion that the presence of bacteria and other microbes indicates an unhealthy or disease situation.^41^ Due to restricted exposure to the outside within each respiratory flow, compensatory capacities of the lungs benefit a relatively steady compositional microbiome in the lower respiratory tract besides terminal alveolus, in which microbial compositions seldomly suffer dynamic alterations and sustain close crosstalk with host epithelial cells instead. These dynamic interactions with the outside make it vulnerable to various microbial interventions from the oral, upper respiratory tract, and gut microbiome with gastric reflux as well.^23^, ^42^ For all that, subtle disturbances in sophisticated environments are adjusted by themselves to maintain the microecological homeostasis within measures in spite of restricted biomass. Thanks to these relatively stable affairs, various microbial and host components in the lower respiratory tract constitute distinct microecological environments together with their corresponding metabolites, seemingly performing as “a private identification card” to distinguish microbial characteristics, health preferences, and underlying disease vulnerabilities over a period of time.^43^, ^44^ In comparison with biopsy‐included traumatic examinations, it presents a practical and relatively convenient potency to launch electronic bronchial environment examinations to verify the specific microbiome, and the corresponding metabolites from the local microecological environment during the diagnostic affairs, drug effectiveness evaluations, and dynamic monitoring of lung cancer with a narrowing distance to clinical practices (Table S7).

It should be noticed that the lower respiratory tract microbiome is of vital importance in shaping a healthy lung immune system, providing resistance to colonization by respiratory pathogens, and as an integral, regulating immune tolerance in the lung microenvironment by balancing tumorigenic inflammation, although great attention has been put on gut microbiome for decades. Theoretically, tumour development constructs an immunosuppressive microenvironment in lung enriched in T regulatory (Treg) cells and M2 macrophages expressing anti‐inflammatory molecules such as programmed death‐ligand 1 (PD‐L1),^11^ which further imbalances anti‐tumoral natural killer (NK) and T cell responses to promote immune tolerance and escape. From this perspective, lung microbiota dysbiosis or the presence of specific microbial strains might contribute to the generation of this immunosuppressive microenvironment and the low efficacy of immunotherapy treatments.^45^, ^46^ As previously reported, *Streptococcus salivarius* and *S. agalactiae* in NSCLC correlated with higher frequencies of T helper type 1 (Th1) and Th17 phenotypes than those in the health, contributing to treatment efficacy.^46^ CD8^+^ T‐cell derived inflammation towards microbes has also been manifested to optimize responses by rebalancing immune responses in vivo, partially reversed by antibiotic aerosolization with immunosuppressive alteration in tumour microenvironment, which is accompanied by the maturation of resident antigen‐presenting cells, rebalance from the activation of the anti‐tumoral response on NK and T cells and the inhibition of pro‐tumoral response on M2 macrophages and Treg cells.^47^, ^48^ However, these trials are described in malignant lesions rather than those in the lower respiratory tract, which are often achieved by traumatic examinations, and further present room for exploring their connections with immune characteristics within BALF fluids instead. Given that, adequate efforts to the association of microbiome, or precisely specific microbes, and anti‐tumoral immune infiltration in the lower respiratory tract together with its underlying mechanisms should be highlighted in a large cohort both from clinical and mechanical perspectives. Thus, we performed a multi‐omics dependent integrated analysis to depict the landscapes of divergent ICI responses with advanced NSCLC and demonstrated a *Bacillus*‐associated inflammatory infiltration as a potential predictor of favoured ICI response.

Apart from direct biofilm contacts or genomic redrafts, microbial metabolic profiles also contribute to the specific immune reconstruction of the lung microenvironment as primary signals by microbe–host crosstalk.^49^ Albeit difficult to achieve up to now, it seems significant to distinguish the origins of altered metabolites from the microbiome or, more possibly, the host. Confronting the same problem with the above, few studies focus on metabolic alterations in the lower respiratory tract but in the bloodstream from gut microbiome with amounts of dominant metabolites associated with ICI responses.^18^ For instance, microbe‐dominant lipid signalling enforced functional specialization of Treg cells to drive immunosuppression by microbial proteins or mitochondrial dependence, which can be reversed by probiotic components from short‐chain fatty acids, a set of intermedium from lipid degradation.^50^ Another, essential amino acids degradation, especially tryptophan metabolism, also matters most in unfavored ICI outcomes. Various studies have approved their negative roles in immune checkpoint blockade efficacies, mainly by improving the escape of immunosurveillance, reconstructing immunotolerance, or them both with microbe‐ or host‐derived byproducts. These products may increase the number of tumour‐infiltrating T cells and other protective anti‐tumoral immune cells as a synergistic partner with PD‐L1 blocking antibodies in a concentration‐ or time‐dependent manner.^20^ Although engineered microbial steps and chemical inhibitory attempts have been put forward preliminarily,^20^ indeed, it still deserves additional concentrations to engage in exploring the underlying mechanisms and applicational potency to enhance immunotherapies efficacy from metabolism perspectives. In this study, we identified the dysfunctional escalation of indicated metabolic pathways in unfavored subgroups, which further associated closely with pathogenic microbes, especially *Sphingomonas* and *Sediminibacterium*, and pro‐tumoral inflammatory factors, emphasizing potential biomarkers in a localized microecological environment to clinical response, and further providing insight for predicting the efficacy of ICIs during clinical practices.

Certainly, the primary limitation of this study seems the shortage of adequate microbial and metabolic characteristics from restricted enrolled participants with advanced NSCLC in this cohort, which may weaken the accuracy of correlational analysis and the prediction model, probably being improved by extended enrollment and additional self‐control before and after ICI interventions in our coming future. Another shortcoming to be noticed is the negligence of the gut microbiome in the presence of the lung‐gut axis as previously discussed, and the latter derived metabolites may exert remote influences via the bloodstream. However, in accordance with the former, participants enrolled retrospectively in this cohort failed to collect faecal samples at the time of BALF collection due to the hysteresis of imaging diagnosis in ICI responses, which could be achieved by a detailed prospective study. Furthermore, detective accuracy of microbiome and metabolome contributed to a relatively mixed background to distinguish the genuine origins from the host or microbes due to the presence of intra‐tumour bacteria as recently discussed. Although representing an improvement upon ICI response prediction models, it is still an emergency to fully describe the microecological characteristics of the lower respiratory tract in favoured ICI responses within advanced NSCLC and, in turn, to develop effectively trained predictors in detail.

## 5 CONCLUSION

In summary, our study described the distinct compositional characteristics of the microecological environment in the lower respiratory tract among different responses to ICI interventions, further identifying 16S rRNA sequencing‐based microbiomes, untargeted metabolome, and immune‐cell derived inflammatory features as potent predictors of responder selection using integrated analyzing approaches. This study demonstrated the potential correlations of these enrolled components, highlighting clinical response predictive potencies from multiple‐dimensional perspectives. Our findings also inspired detailed investigations into the practical applications of microecological characteristics‐driven markers in ICI therapeutic regimes by bronchoalveolar lavage fluids.

## AUTHOR CONTRIBUTIONS

Yong Zhang: Project administration, formal analysis, and roles/writing—original draft. Xiang‐Xiang Chen: Methodology and validation. Ruo Chen: Validation and project administration. Ling Li: Methodology and validation. Qing Ju: Resources and visualization. Dan Qiu: Data curation. Yuan Wang: Investigation. Peng‐Yu Jing: Resources and validation. Ning Chang: Resources. Min Wang: Resources. Jian Zhang: Supervision and funding acquisition. Zhi‐Nan Chen: Supervision and writing—review and editing. Ke Wang: Conceptualization, supervision, funding acquisition, and writing—review and editing.

## CONFLICT OF INTEREST STATEMENT

The authors declare no conflict of interest.

## SUPPORTING INFORMATION

Additional supporting information can be found online in the Supporting Information section at the end of this article.

## Supporting information



Supporting Information

Supporting Information

Supporting Information

## Data Availability

All data generated or analyzed in this study were oriented from a standardized clinical process and included in this published article. Sequence data that support the findings of this study should be directed to and will be fulfilled by the lead contact Ke Wang at wangke@fmmu.edu.cn.
