# Supplementary material for: Lower respiratory tract microbiome dysbiosis impairs clinical responses to immune checkpoint blockade in advanced non‐small‐cell lung cancer
Source: Clin Transl Med. 2025 Jan 10;15(1):e70170. doi: 10.1002/ctm2.70170 (PMC11726686; doi:10.1002/ctm2.70170)
Supplement: Supplementary file 1 — Supporting Information [file CTM2-15-e70170-s001.docx]

**Supplementary material tables**

| **REAGENT or RESOURCE** | **SOURCE** | **IDENTIFIER** |
| --- | --- | --- |
| **Biological samples** |  |  |
| Human bronchoalveolar fluids | This study | N/A |
| Human blood serum | This study | N/A |
| Malignant tissue sections | This study | N/A |
| **Antibodies and commercial assays** |  |  |
| CD4 Rabbit mAb | CST | Cat#48274s; Clone: EP204 |
| CD8α Rabbit mAb | CST | Cat#85336s; Clone: D8A8Y |
| CD68 Rabbit mAb | Abcam | Cat#ab213363; Clone: EPR20545 |
| CD206/MRC1 Rabbit mAb | CST | Cat#24595s; Clone: E6T5J |
| Opal 6-Plex Manual Detection Kit | AKOYA | NEL811001KT |
| LEGENDplex Human Inflammation Panel 1 | BioLegend | Cat#740808 |
| **Software and algorithms** |  |  |
| R software | R Development Core Team | https://cran.r-project.org/ |
| Python | Python Software Foundation | https://www.python.org/ |
| Bowtie2 | Bowtie2 Software | https://anaconda.org/bioconda/bowtie2 |
| mOTUs2 | mOTUs2 Software | http://motu-tool.org/ |
| HUMAnN2 | HUMAnN2 Software | https://huttenhower.sph.harvard.edu/ humann2/ |
| Microsoft Excel | Microsoft software | https://www.microsoft.com/zh-cn/ |
| Origin | OriginLab | https://www.originlab.com/ |
| GraphPad Prism | GraphPad software | www.graphpad.com/scientific-software/prism/ |
| Omicsmart | GeneDenovo | http://www.omicsmart.com/ |
| BioRender | BioRender Online | https://www.biorender.com/ |
