# Supplementary material for: Lower respiratory tract microbiome dysbiosis impairs clinical responses to immune checkpoint blockade in advanced non‐small‐cell lung cancer
Source: Clin Transl Med. 2025 Jan 10;15(1):e70170. doi: 10.1002/ctm2.70170 (PMC11726686; doi:10.1002/ctm2.70170)
Supplement: Supplementary file 2 — Supporting Information [file CTM2-15-e70170-s003.docx]

**Additional file 1: Figures**

**Supplementary Figure 1**


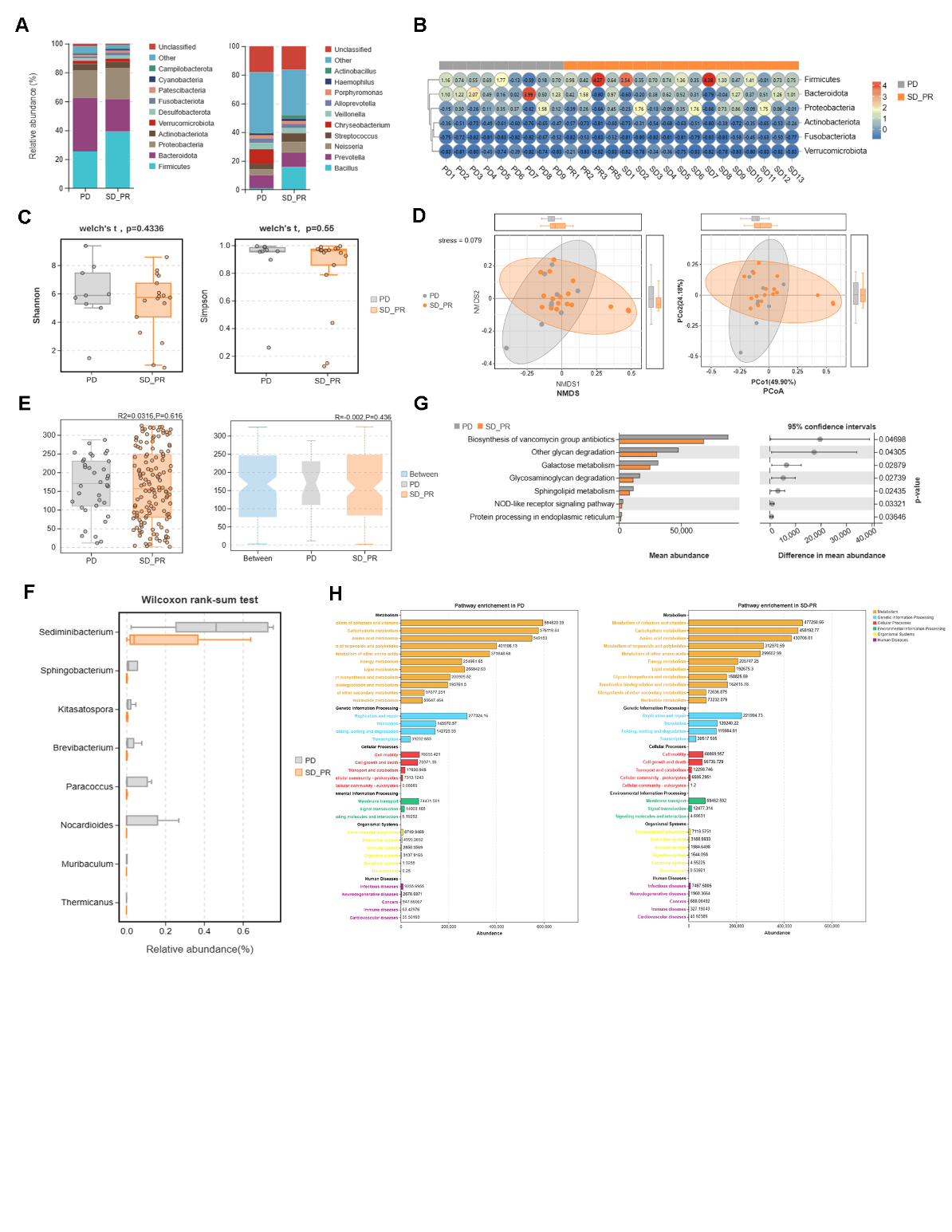


# Fig.S1 Microbial profile alterations among lower respiratory tracts to ICB response in advanced NSCLC. Related to Fig.2. (A) Microbial taxonomy at phylum (left) and genus (right) level in NR and R. (B) Heatmap showing the dominant phyla within each patient of indicated subgroups based on normalized relative abundance over 10% in at least 10 samples according to Z-score. (C) Alpha diversity measured by inverse Shannon (left) and Simpson (right) index of both groups using Welch’s t test. *P* values are shown on the top. (D) Non-metric multidimensional scaling (NMDS, left) and principal Coordinate analysis (PCoA, right) of beta diversity measurements based on Bray-Curtis distances for PD, SD, and PR. Each symbol represented one individual patient. (E) Adonis (left) and Anosim (right) showing the outcomes of beta diversity measurements based on Bray-Curtis distances for NR and R. R^2^ and *P* values are shown on the top. (F) Histogram showing the microbial discrepancy among the both groups using Wilcoxon rank-sum test (FDR<0.05, ^*^*P*<0.05). (G) Histogram of microbial function diversity of indicated subgroups based on mean abundance with 95% confidence intervals. Pathways with ^*^*P*<0.05 are listed on the right. (H) Bar charts showing the relative abundance of NR (left) and R (right) on KEGG pathways in PICRUSt2 database.

**Supplementary Figure 2**


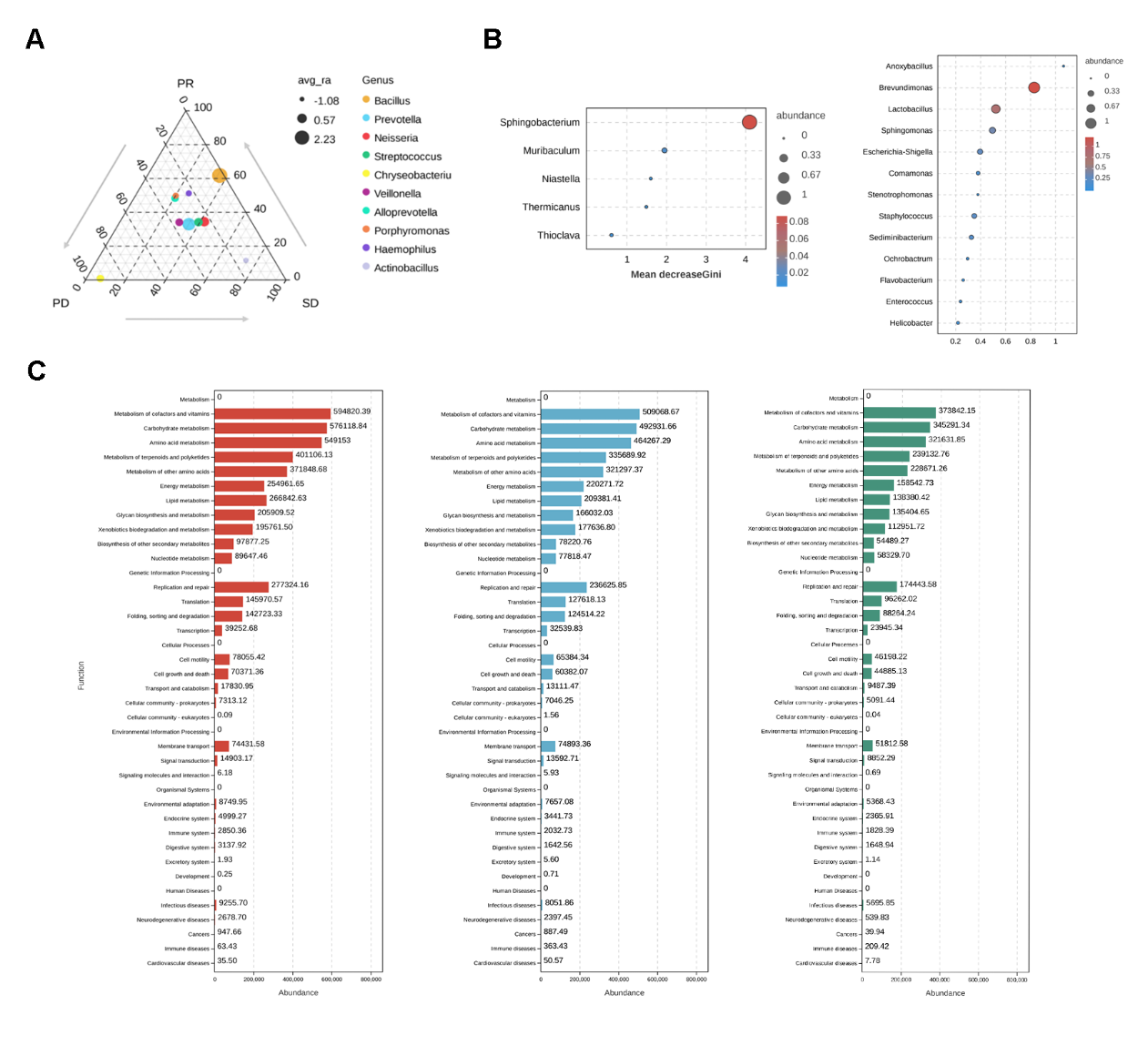


**Fig.S2 Compositional microbial diversity and corresponding functional characteristics of microbial pathways. Related to Fig.3.** (**A**) Ternary plot showing the compositional diversity of microbes in PD, SD, and PR subgroups. (**B**) Random forest showing the indicating genera among PD vs. PR based on mean decrease Gini. The size and color of bubbles represent the abundance of corresponding genera, while the position of bubbles represents the size of the index. (**C**) Bar charts showing the relative abundance of PD (left), SD (medium) and PR (right) on KEGG pathways in PICRUSt2 database, respectively.

**Supplementary Figure 3**


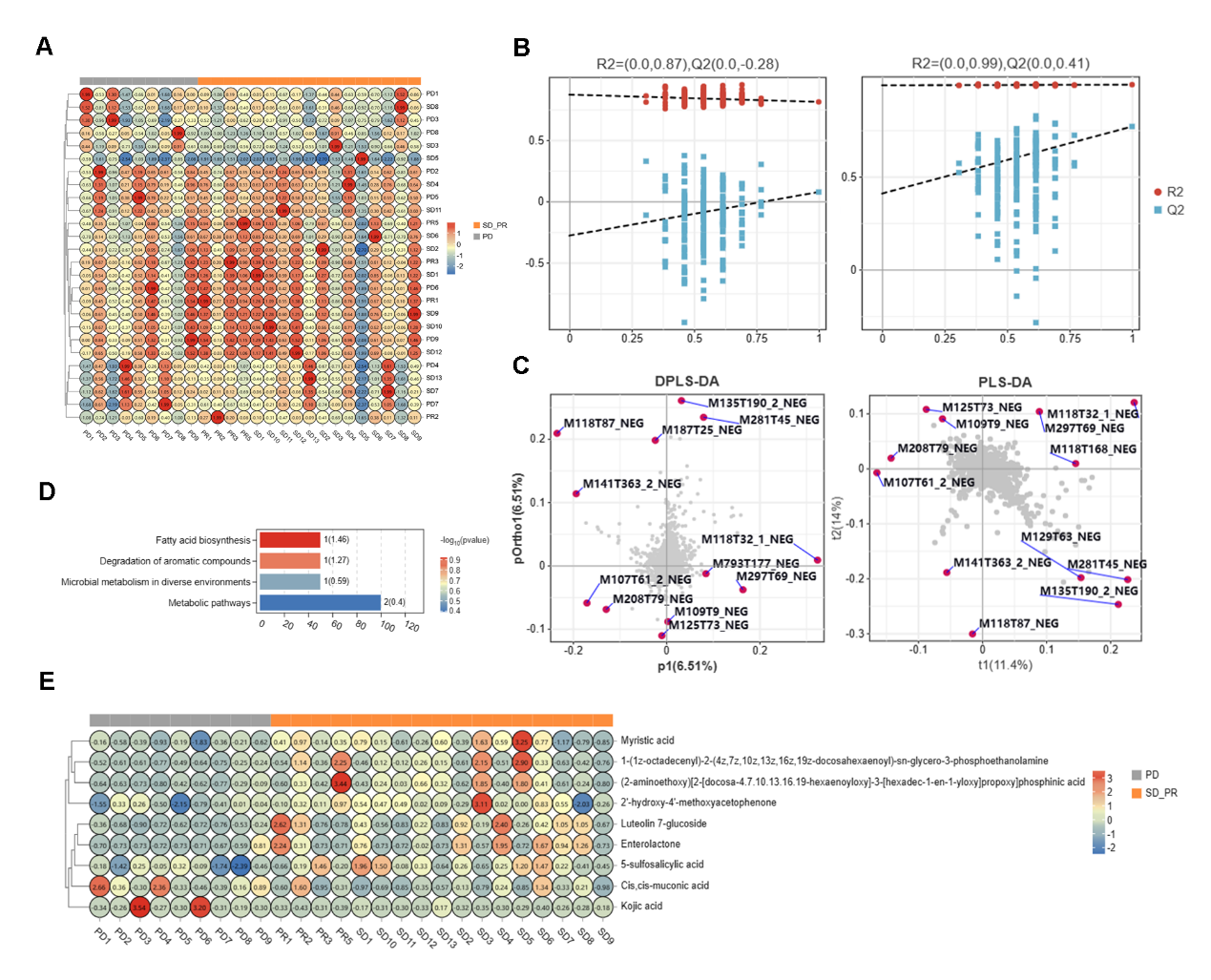


**Fig.S3 Metabolic profiles and their corresponding verifications in NR and R subgroups. Related to Fig.4.** (**A**) Heatmap showing the degree of variation in metabolite composition and abundance between samples quantitatively analyzed through Pearson correlation data at corresponding sites. (**B**) Permutation test showing the accuracy of OPLS-DA (left) and PLS-DA (right) model, respectively. (**C**) Loading diagrams showing the variable with the highest principal component contribution within NR and R subgroups in OPLS-DA (left) and PLS-DA (right) model, respectively. Differently enriched metabolites are marked with Compound ID. (**D**) Bar chart showing the top enriched pathways in KEGG pathways compared with NR and R subgroups. Q values adjusted to -log10 according to enrichment degree. FDR<0.05, ^*^*P*<0.05. (**E**) Heatmap showing the Spearman correlations of candidate differential metabolites with each enrolled patient. *P* values are marked at corresponding sites.

**Supplementary Figure 4**


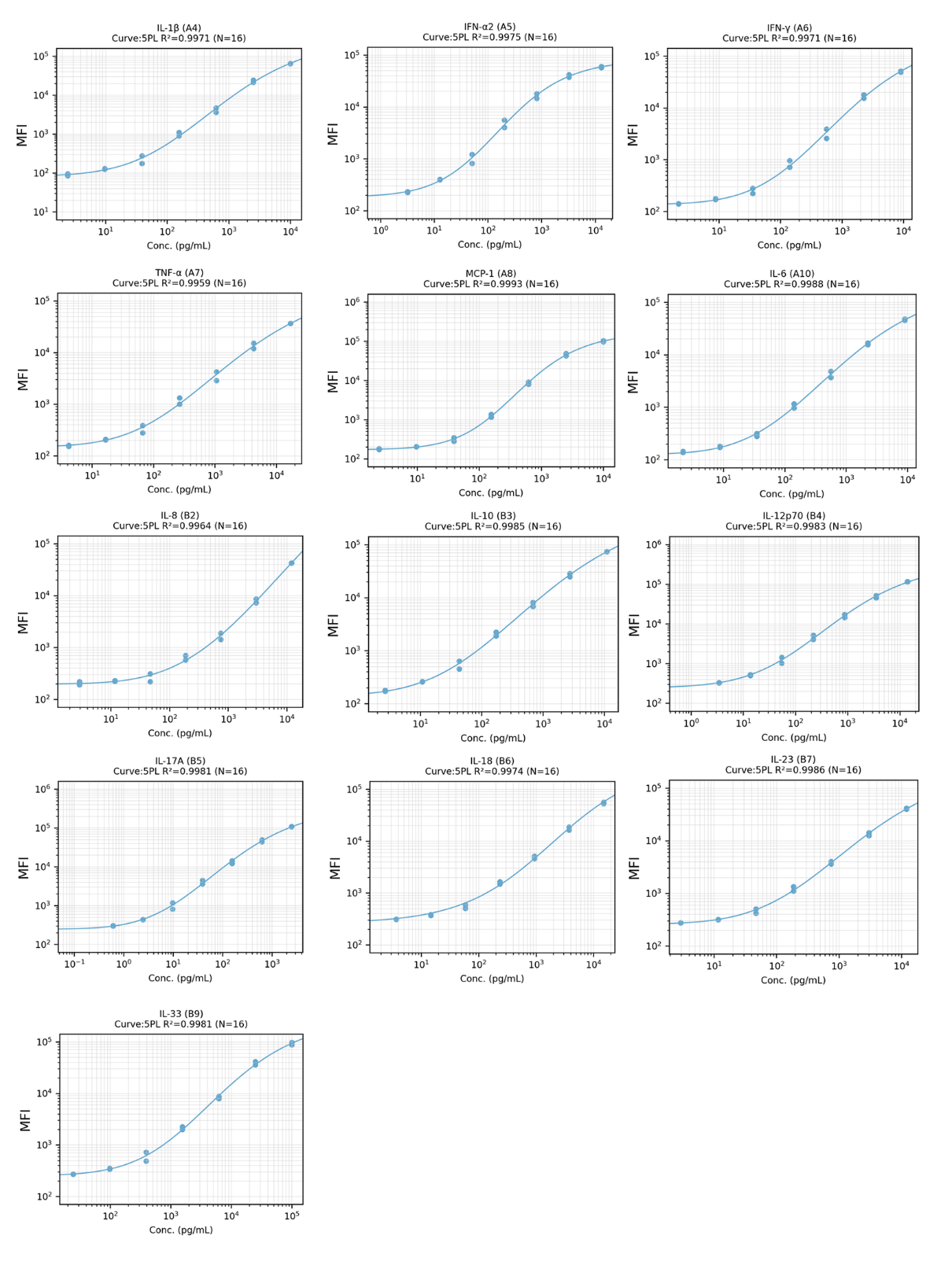


**Fig.S4 Standard curves of 13 cytokines and chemokines detected by Biolegend products using bead-assisted multiplex cytokine profiling. Related to Fig.5.** Candidate cytokines and chemokines in LEGENDplex were detected using Multi-Analyte Flow Assay Kit, Human Inflammation Panel 1 (13-plex) with Filter Plate. Standard curves of each cytokine and chemokine showing the demonstration purposes to guarantee the sensitivity and accuracy by FCS file-targeted 5 parameter fitting analysis from Biolegend’s LEGENDPLEX data analysis software ([www.biolegend.com/en-us/legendplex](http://www.biolegend.com/en-us/legendplex)). Bracketed number in the title equals to unified Beads ID in Biolegend database. Repeated columns are also listed after R^2^ from BALFs and serum, respectively.

**Supplementary Figure 5**

**
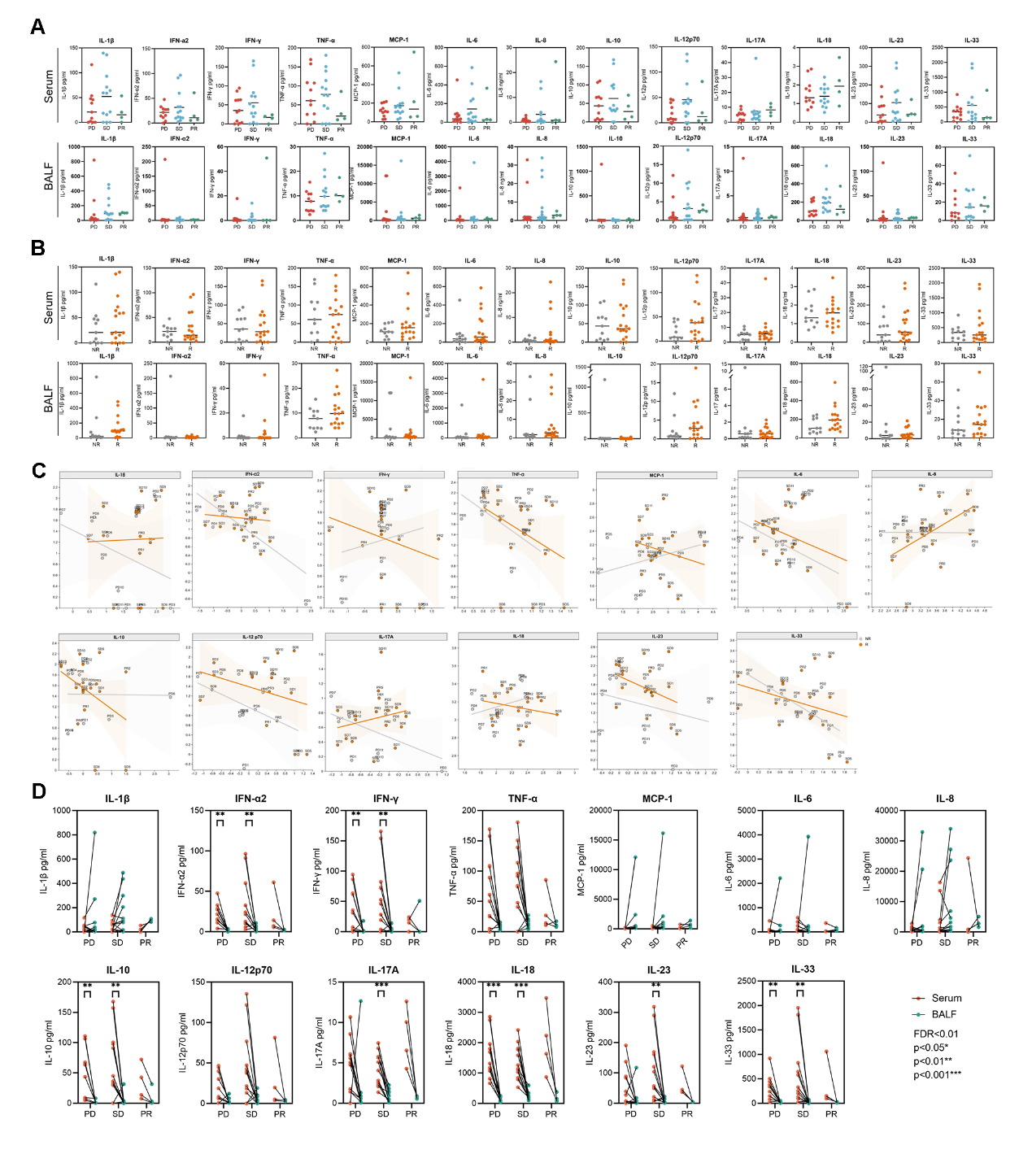
**

**Fig.S5 Correlations and discrepancy of indicated inflammatory analytes in BALFs and serum samples among different subgroups. Related to Fig.5.** (**A**) Bar charts showing the compositional diversity of inflammatory candidates in BALFs and serum within PD, SD, and PR subgroups, respectively. (**B**) Bar charts showing the compositional diversity of inflammatory candidates in BALFs and serum within NR and R subgroups, respectively. (**C**) Linear correlations of indicated inflammatory analytes from BALFs and serum NR and R. (**D**) Compositional diversity and potent correlation of indicated inflammatory analytes in BALFs and serum within different ICB responses. Two groups comparison applied to Wilcoxon rank sum test (two side comparison, FDR<0.05), and three groups comparison to Kruskal Wallis rank sum test (two side comparison). ^*^*P*<0.05, ^**^*P*<0.01, ^***^*P*<0.001, ns, no significance.
