# Supplementary material for: Lower respiratory tract microbiome dysbiosis impairs clinical responses to immune checkpoint blockade in advanced non‐small‐cell lung cancer
Source: Clin Transl Med. 2025 Jan 10;15(1):e70170. doi: 10.1002/ctm2.70170 (PMC11726686; doi:10.1002/ctm2.70170)
Supplement: Supplementary file 3 — Supporting Information [file CTM2-15-e70170-s002.docx]

**Additional File 2: Tables**

**Table S1. Clinical Manifestation of Advanced NSCLC Patients Receiving Immune Checkpoint Inhibitor. Related to Figure 1.**

**Table S2. Univariate and Multivariate Analysis for PFS in the NSCLC Cohort. Related to Figure 1.**

**Table S3. Differentiated Candidate Microbes According to LEfSe Analysis Among These Three Groups. Related to Figure 2.**

**Table S4. Receiver Operating Characteristic (ROC) Curves of Top Bacterial Identifiers within Respective Groups**

**Table S5. Pearson Linear Regression of Indicated Cytokines and Chemokines in BALF and Serum**

**Table S6. Spearman Correlation of Indicated Cytokines and Chemokines in BALF and Serum**

**Table S7. Ongoing Clinical Trials on The Association Between Microbiome and Non-Small Cell Lung Cancer**
